# The *NBN* founder mutation—Evidence for a country specific difference in age at cancer manifestation

**DOI:** 10.1002/cnr2.1700

**Published:** 2022-08-10

**Authors:** Krystyna H. Chrzanowska, Eva Seemanova, Raymonda Varon, Martin Digweed, Dorota Piekutowska‐Abramczuk, Karl Sperling, Pavel Seeman

**Affiliations:** ^1^ Department of Medical Genetics The Children's Memorial Health Institute Warsaw Poland; ^2^ Department of Clinical Genetics, Institute of Biology and Medical Genetics, 2nd Medical School Charles University Prague Czech Republic; ^3^ Institute of Medical and Human Genetics Charité‐Universitätsmedizin Berlin Berlin Germany; ^4^ DNA Laboratory, Department of Pediatric Neurology, 2nd Medical School Charles University, University Hospital Motol Prague Czech Republic

**Keywords:** age of cancer manifestation, cancer risk of heterozygotes, environmental and medical exposure to ionizing radiation, NBS

## Abstract

**Background:**

Nijmegen breakage syndrome (NBS) is an autosomal‐recessive chromosome instability disorder characterized by, among others, hypersensitivity to X‐irradiation and an exceptionally high risk for lymphoid malignancy. The vast majority of NBS patients is homozygous for a common Slavic founder mutation, c.657del5, of the *NBN* gene, which is involved in the repair of DNA double‐strand breaks (DSBs). The founder mutation also predisposes heterozygous carriers to cancer, apparently however, with a higher risk in the Czech Republic/Slovakia (CS) than in Poland.

**Aim:**

To examine whether the age of cancer manifestation and cancer death of *NBN* homozygotes is different between probands from CS and Poland.

**Methods:**

The study is restricted to probands born until 1989, before replacement of the communist regime by a democratic system in CS and Poland, and a substantial transition of the health care systems. Moreover, all patients were recruited without knowledge of their genetic status since the *NBN* gene was not identified until 1998.

**Results:**

Here, we show that cancer manifestation of *NBN* homozygotes is at a significantly earlier age in probands from CS than from Poland. This is explained by the difference in natural and medical radiation exposure, though within the permissible dosage.

**Conclusion:**

It is reasonable to assume that this finding also sheds light on the higher cancer risk of *NBN* heterozygotes in CS than in Poland. This has implications for genetic counseling and individualized medicine also of probands with other DNA repair defects.

## 1 INTRODUCTION

Nijmegen breakage syndrome (NBS [MIM: 251260]) is an autosomal‐recessive chromosome instability disorder characterized by microcephaly, growth retardation, immunodeficiency, hypersensitivity to X‐irradiation and an exceptionally high risk for lymphoid malignancy. The latter is the major cause of early death among NBS patients.^1^ The majority of NBS patients originates from Eastern Europe and is homozygous for a common Slavic founder mutation in the *NBN* gene coding for nibrin, c.657del5.^2^ The protein nibrin is part of the trimeric MRE11/RAD50/NBN complex involved in the repair of DNA double‐strand breaks (DSBs) and their processing during immune gene rearrangements, telomere maintenance, and meiotic recombination.^3^, ^4^, ^5^, ^6^

A high frequency of heterozygous c.657del5 carriers has been reported from Poland and the Czech Republic/Slovakia (abbreviated as CS). The founder mutation also predisposes heterozygous carriers to cancer,^7^ which was rigorously confirmed in CS on blood relatives from NBS patients between 1998 and 2003 whose carrier status was unknown at the time of recruitment. Among the 141 carriers (ø age 48 years) were 17 cases of cancer.^8^ In parallel 74 Polish carriers of the founder mutation were recruited until 2002 (ø age 45 years) with only two cases of cancer.^9^ Moreover, in two independent studies the frequency of carriers of the *NBN* c.657del5 mutation was analyzed in adult cancer patients from Poland and CS in comparison to a control population. The relevant odds ratios are 1.9 (Poland)^10^ and 3.1 (CS).^11^ These findings similarly point to a lower cancer risk of *NBN* carriers in Poland compared to CS. This prompted us to look at an independent parameter: The age at cancer manifestation in *NBN* homozygotes from both countries. The analysis is restricted to probands born before replacement of communism by democracy in 1989; in June in Poland and in November in CS. This led to a substantial transition of the health care systems in both countries.^12^, ^13^ Moreover, all patients were born before identification in 1998 of the *NBN* gene responsible for the chromosomal instability syndrome. Thereafter, treatment and preventive care of the patients changed completely due to their extreme radiosensitivity. Here, we show that cancer manifestation of *NBN* homozygotes is at a later age in probands from Poland than from CS.

## 2 PATIENTS AND METHODS

The study is based on 23 *NBN* homozygotes from Poland and 21 from CS. For more than 20 years, Krystyna H. Chrzanowska and Eva Seemanova have supervised the clinical management of homozygous patients with NBS in both countries. To the best of our knowledge the Polish and CS probands comprise all cases ascertained by Krystyna H. Chrzanowska and Eva Seemanova until 1989. Nonetheless, underdiagnosis of NBS cannot be excluded because probands who died from cancer at an early age could be overlooked. Until 1989, medical care in both countries was provided according to uniform, country‐specific criteria. After replacement of the communist system, significant changes took place in medical care in both countries. Moreover, until 1989 there was no knowledge of the particular radiation sensitivity of the subjects. This changed fundamentally with the detection of the founder mutation in 1998. Since then, diagnosis was made shortly after birth in Poland and CS and accompanied by appropriate recommendations to avoid radiation risks. Molecular testing after 1998 confirmed that all clinically diagnosed NBS patients were homozygous for the *NBN* founder mutation by PCR of exon 6 of the *NBN* gene and fragment analysis^14^ or Sanger sequencing.

Statistical analyses were carried out with the Mann–Whitney *U* Test.

The study was performed with the ethical approval of The Children's Memorial Health Institute, Warsaw and the Ethics Committee of the University Hospital Motol and the 2nd Medical School of the Charles University, Prague.

## 3 RESULTS AND DISCUSSION

### 3.1 Cancer occurrence in NBS patients

In Poland 23 *NBN* homozygotes, born between 1962 and 1989, were identified, 12 males and 11 females. Seventeen of these patients developed cancer: 15 of lymphoid origin, 1 a medulloblastoma, and 1 a thyroid carcinoma. The age at cancer manifestation varied between 7 and 35 years, 3 probands were unaffected at the age of last contact when they were 37, 39, and 42 years old. The latter two cases had normal IgG and IgA levels (Table 1). The birth places of the probands are located in the Polish territory that belongs to the North European lowlands, covered by glacial deposits (Figure S1A).

**TABLE 1:** *NBN* homozygotes from Poland born until 1989 and age at cancer manifestation

*N*	ID	Sex	Year of birth	Cancer	Age at cancer manifest	Age at death	Age at last contact	Ig‐status
								Age (years)
1	B‐5567	m	1962	T‐NHL	34	34		nd
2	B‐3199	m	1977	T‐NHL	24	27		IgG↓; IgA↓
				DLBCL	27			14 6/12
3	B‐4783	f	1978	B‐NHL	15	21		IgG↓; IgA↓
								10
4	B‐3775	f	1978				42	IgG+; IgA+
								14 1/2
5	B‐5450	f	1978				39	IgG+; IgA+
								18
6	B‐3197	f	1979	Lymphoma	15	15		IgG↓; IgA+
								13 9/12
7	B‐3205	m	1980	DLBCL‐1‐5	11	29		IgG+; IgA+
								10 3/4
8	B‐3197	m	1981	ALL‐T	35	36		IgG↓; IgA+
								11 6/12
9	B‐3199	m	1981				37	IgG↓; IgA↓
								10
10	B‐3220	m	1982	TLBL/ALL	16	19		IgG↓; IgA↓
								10 7/12
11	B‐6019	m	1983	T‐NHL	11	12		nd
12	B‐6646	f	1985			23		nd
13	B‐6023	m	1985	B‐NHL	7	19		IgG+; IgA+
								7,5
14	B‐3258	m	1985	Thyroid Ca	20		35	IgG↓; IgA+
				NHL (MCL)	35			9
15	B‐4290	f	1985	Medulloblastoma	8	9		IgG↓; IgA↓
								8 9/12
16	B‐5345	m	1986	HL	12	14		IgG↓; IgA↓
								6 1/2
17	B‐3426	f	1987	B‐NHL	9	9		IgG↓; IgA↓
								7
18	B‐3201	f	1987	T‐NHL	19	21		IgG+; IgA+
								5 1/2
19	B‐5700	f	1988	T‐NHL	24	24		IgG↓; IgA↓
								8 1/2
20	B‐4285	f	1988	B‐NHL	12	13		IgG↓; IgA↓
								6 1/2
21	B‐3432	m	1989			13		IgG↓; IgA↓
								4
22	B‐5337	f	1989	TLBL/ALL	9	12		IgG+; IgA+
								7
23	B‐3316	m	1989			8		IgG↓; IgA↓
								4

Abbreviation: nd, no data.

In CS, 21 *NBN* homozygotes, born until 1989, were recruited. Cancer has been confirmed in 17 of them: 13 of lymphoid origin, one medulloblastoma, gonadoblastoma, meningioma, and Ewing sarcoma each. The age at cancer manifestation varied between 1 and 24 years (Table 2). The map with the locations of the CS probands illustrate that they are widely distributed over CS (Figure S1B).

**TABLE 2:** *NBN* homozygotes from CS born until 1989 and age at cancer manifestation

*N*	ID	Sex	Year of birth	Cancer	Age at cancer manifest	Age at death	Age at last contact	Ig status
								Age (years)
1	MD	f	1969	NHL	10	10		nd
2	PJ	m	1970	ALL	10	10		nd
3	DM	f	1971	NHL	18	22		nd
4	ZZ	f	1973	Gonadoblastoma	17	19		nd
5	VS^a^	f	1973	‐			32	nd
6	CJ	m	1975	NHL	24	29		nd
7	DD	m	1977	NHL	2	2		nd
8	PA	f	1978	ALL	1	1		nd
9	CR	f	1979	Ewing Sarcoma	11	17		IgG↓
								15
10	ZR	f	1979	Meningioma	10		26	IgG↓; IgA↓
								7
11	AP	m	1980	ALL Hodgkin	13 17		25	IgG+; IgA+
								16 1/2
12	ZL	f	1980	‐		0.5		nd
13	ZJ	m	1981	NHL	9	10		nd
14	PJ	f	1983	Medulloblastoma	7	7		nd
15	HM	m	1984	‐			21	nd
16	SJ	f	1985	NHL	16		20	nd
17	DL	f	1986	NHL	7	10		IgG↓; IgA↓
								5
18	CM	m	1988	NHL	9	10		nd
19	FM	f	1989	‐			21	IgG↓; IgA↓
								7
20	HL	m	1989	ALL	6	13		nd
21	OM	m	1989	NHL	10	16		nd

Abbreviation: nd, no data.

^a^ Proband without any diagnostic X‐irradiation.

The Polish cases 2 and 9, 6, and 8 are siblings. There was no evidence of consanguinity, in two cases (3 and 22) the ancestors came from closely neighboring places. The parents of the CS probands 8 and 17 are first cousins and five pairs are siblings (2 and 8, 3 and 7, 6 and 9, 12 and 13, 15 and 20). Since the sibs do not represent independent observations, we tested whether this has an influence on cancer manifestation. We calculated the difference of age in cancer manifestation between the siblings and between unrelated subjects. For the latter, we simply compared two consecutive individuals listed in Tables 1 and 2. The age difference was 14.5 years for the siblings and 7 years for unrelated individuals (Tables S1 and S2). Clearly, the number of cases is small, but it is evident that there is no tendency for greater concordance in age at cancer manifestation among the siblings.

Both cohorts match with regard to age distribution relative to the year 1990 (*p* = .13, Mann–Whitney *U* Test). The average age at cancer manifestation, however, was at 16.5 years significantly higher in Poland than in CS at 10.6 years (*p* = .03, Mann–Whitney *U* Test). Similarly, age at death was significantly different—also higher in Poland (*p* = .04, Mann–Whitney *U* Test). The age at diagnosis of NBS in CS and Poland with 7.1 and 7.2 years corresponded rather well, however, 37% of the children from CS had already lymphoma in contrast to 14% of Polish children.^15^

Before the *NBN* gene was identified, patients from the Netherlands^16^ and the Czech Republic^17^ had been assigned to two different autosomal‐recessive genetic disorders with microcephaly, a characteristic face, and immunodeficiency in common. The main difference was the high risk for early manifestation of lymphoreticular malignancies observed in the CS patients. Here, we demonstrate that tumor manifestation is also earlier in CS than in Polish patients with the same Slavic founder mutation.

### 3.2 DNA repair and lymphoid malignancies in NBS

In both cohorts the vast majority of malignancies are of lymphoid origin. These cells differ from all other cells in their hypermutability due to somatic recombination of the immunglobuline and T‐cell receptor genes necessitating DNA‐DSBs and their subsequent repair. As the *NBN* gene is involved in this process, the particularly high lymphoma risk is not surprising. B‐ and T‐cell lymphomas occur equally often. The risk for developing lymphoma is increased to over 1000‐fold for NBS patients.^18^ In the general population, the majority of pediatric lymphomas stem from B‐cells (B‐NHL and Burkitt lymphomas), in adults diffuse large B‐cell lymphoma (DLBCL) predominate. The spectrum of lymphoproliferative disease in NBS patients is more characteristic for adult rather than pediatric patients and obviously reflects a different mechanism of lymphomagenesis.^18^ Moreover, the development of B‐ and T‐cell lymphoma is highly increased in immunodeficient patients, also a characteristic of *NBN* homozygotes (Tables 1 and 2). The concordance of tumor types between the CS and Polish probands makes it rather unlikely that diagnostic differences are responsible for the age difference in cancer manifestation. This is also supported by the high correlation in the time between cancer manifestation and death between both two cohorts (*p* = .9, Mann–Whitney *U* Test).

The involvement of the *NBN* gene in the repair of DSBs also explains the high sensitivity of *NBN* homozygotes to ionizing radiation and radiomimetics. This is indicated by the rapidly progressing organ insufficiency and death after radiotherapy and the improvement of life expectancy after introduction of appropriate preventive care, such as avoiding oxidative stress.^1^ In vitro, the radiation sensitivity is indicated by the increase in cell death, the reduction in colony forming assays, and the drastically increased frequency of chromosome aberrations following irradiation.^3^, ^19^, ^20^, ^21^

Generally, the diseases with a DNA repair defect confirm the role of an increased mutation rate in the multistep nature of carcinogenesis. However, epigenetic effects should also be kept in mind. Thus, low dose irradiation (10 cGy) of human reproductive cells in vitro with no influence on cellular viability can induce epigenetic alterations that affect, among others, the expression of gene clusters related to the DNA damage response in somatic and cancer cells.^22^

### 3.3 Comparison with other studies on cancer in NBS

Interestingly, in the largest international study of 226 patients with NBS, diagnosed between 1993 and 2018,^23^ the cumulative incidence of cancer in NBS was considerably higher than reported in previous studies.^1^ For the large Polish cohort with 133 patients it was 72%, for the non‐Polish cohort, 88%. In our study the corresponding data for Poland and CS are 74% and 81%, respectively. Interestingly, an initial rapid phase of cancer increase was noted until the age of 7 years, but significantly slower in the Polish cohort. In our cohorts, only one Polish proband manifested cancer until the age of 7 but five from CS. Thereafter, the average age of cancer manifestation is with 17.1 years for the Polish probands later than for the CS probands with 13.1 years underlining that the country specific effect is obviously not confined to the phase of early cancer manifestation. It should be noted that the difference in the initial phase between the two cohorts of the international study was discussed with respect to different diagnostic strategies, genetic counseling or varied familial clustering patterns.

Clearly, these aspects also limit a comparison with other multicenter NBS studies, such as that of Sharapova et al.^24^ on 136 patients, recruited between 1983 and 2018, with a focus on regional differences in cancer manifestation or by Deripapa et al.^25^ on 35 Russian NBS patients enrolled from 2012 to 2016, a time when the therapeutic measures were also very different compared to our cohort. With the reductionist approach of our study, the limitation to the year of birth until 1989 and the registration and care of the patients by two designated specialists, an attempt was made to avoid the above “recording bias” as far as possible.

### 3.4 Potential ascertainment bias in determining age at cancer manifestation

Concerning the age difference in cancer manifestation, both ascertainment bias as well as exogenic and endogenic factors have to be kept in mind. Before DNA testing was possible, the age of correct diagnosis of *NBN* homozygotes was around 7 years in both CS and Poland.^15^ After identification of the founder mutation in 1998, the age of diagnosis dropped to less than 1 year in CS.^18^ This was the prerequisite for preventive care through complete avoidance of exposure to X‐rays and other mutagens. In the present context it is important that all probands were registered before their *NBN* genotype was known, including the two CS probands who developed cancer at the age of 1 and 2 years. In addition, a tumor cannot be ruled out in CS case 12, who already died at the age of 0.5 years, as no necropsy was performed. Thus, due to NBS underdiagnosis at that time an ascertainement bias with respect to the registration of NBS homozygotes who passed away at an early age cannot be excluded a priori.

In this context it is relevant that the long‐term study of 133 Polish NBS patients from birth to adulthood gave no evidence of exitus before 2 years of age.^23^ Moreover, prenatal lethality in NBS families is even lower than in the general population. Thus, there is no evidence for an ascertainment bias.

Clearly, the number of cases is limited, their genetic constitution, however, is exceptional: All are homozygous for the Slavic founder mutation *NBN* c.657del5, which occurred before less than 300 generations.^26^ In the fifth and sixth century, during the early medieval waves of migration into Europe, the Slavs split into the Southern, Eastern, and Western Slavs. The latter include the Czechs, Slovaks, and Poles. They are closely related to each other and it is therefore rather unlikely that the difference in cancer risk is due to modifying genes enriched in one population. Thus, if one accepts that the observed difference in the age of cancer manifestation in *NBN* homozygotes between CS and Polish probands is not due to chance or ascertainment bias an exogenic, environmental cause should be taken into account.

### 3.5 Exogenic causes to explain differences in age at cancer manifestation

After the Second World War, both Poland and CS implemented a more centralized health care model based on Soviet experience. Since the 1970s, cardiovascular diseases were the major cause of death, followed by malignant neoplasms. In both countries, tobacco and alcohol consumption were highly relevant risk factors.^12^, ^13^ There is, however, a third risk factor with differences between both populations: exposure to natural and man‐made ionizing radiation.

**FIGURE 1:**
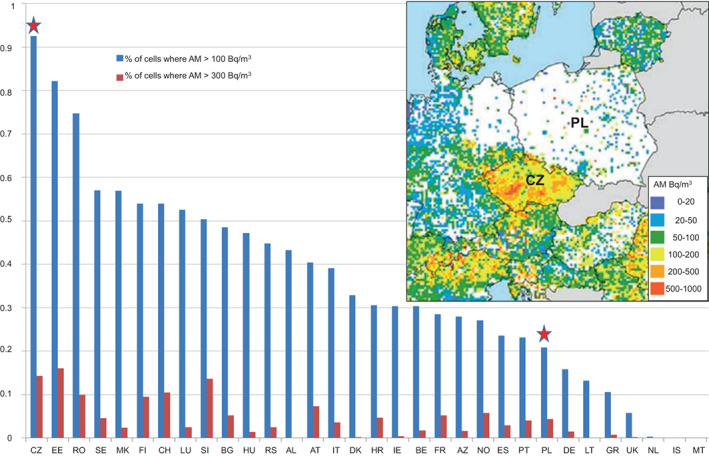
Indoor radon concentrations in 32 European countries, estimated in 10 × 10 km cells. Given is the percentage of cells with an arithmetic mean (AM) above 100 and 300 Bq/m^3^.^27^ Inset: Map of estimated indoor radon concentrations across Europe, especially Czechia (CZ) and Poland (PL). The doses were estimated in 10 × 10 km cells and depicted as arithmetic means^28^

Indoor radon is the most important contributor to population radiation dose and its concentration essentially reflects the underlying geology. In Europe, high radon concentrations are found in the granitic areas of the Bohemian Massif. Based on *The European Atlas of Natural Radiation* ^27^ the level of natural radioactivity among 32 European countries is highest in the Czech Republic while Poland ranks only 25th (Figure 1). Unfortunately, no data are available for Slovakia.

The population‐weighted, average annual effective dose for all external and internal natural radiation sources is 5.83 mSv for the Czech republic and thus higher than the 2.77 mSv for Poland.^28^

In addition, medical exposure due to diagnosis must be taken into account. Retrospective assessment of the received doses of ionizing radiation is almost impossible without knowledge of the radiologic equipment and the setting for in situ dosimetric measurements. Clearly, the exposure before 1989 was higher than today due to improvements in radiologic techniques and increased concern about radiation protection.

Irrespective of this, there is indirect evidence that diagnostic X‐ray examinations played a greater role in the medical care of the population in CS than in Poland. In the period from 1952 to 1992, regular chest X‐ray diagnostics was mandatory for the entire population in CS in order to prevent the spreading of tuberculosis. Similarly, X‐ray examinations to diagnose hip dislocation was common. The chest X‐ray screening was performed by abreography, which has an about 10x higher dose of irradiation than a regular chest X‐ray examination. Pregnant women also had their lungs X‐rayed in the first trimenon. All children at the age of 2 months had their hips and pelvis radiographed. The Czech Republic is even today better equipped with diagnostic imaging technologies than the other Visegrád group countries.^13^ In contrast, the Polish medical system was suffering from heavy losses in the number of healthcare providers. The total number of diagnostic X‐ray examinations per 1000 inhabitants was 572 in 1986, much less than in the Czech Republic.^29^

According to the United Nations Scientific Committee on the Effects of Atomic Radiation (UNSCEAR) Report^30^ the utilization of X‐rays for diagnosis in medicine varied significantly between countries. Unfortunately, detailed information is available only for Czechoslovakia and the Czech Republic (Cz), but not for Poland (Po). However, for both countries information on diagnostic nuclear medicine procedures are presented (Table 3).

**TABLE 3:** Diagnostic nuclear medicine procedures in the Czech Republic (Cz) and in Poland (Po) according to the UNSCEAR Report^30^

Diagnostic nuclear medicine procedures	Cz	Po
Annual number of diagnostic nuclear medicine procedures per 1000 population from 1997 to 2007	12.8	3.0
*Diagnostic nuclear examinations per million population*		
Renal	1635	431
Gastroenterology	1090	26
Brain	570	68
Number of PET or PET‐CT scanner per million population	0.29	0.05
Number of relevant physicians per million population	15	3.9
Number of radiotherapy centers per million population	3.7	0.6

Considering these data, it is realistic to assume that before the 1989 revolution diagnostic irradiation was considerably higher in CS than in Poland. In this context, one case report might be relevant: The oldest living CS NBS patient (no. 5 in Table 2) is from Eastern Slovakia and has had, in contrast to all other CS patients, no radiological examinations at all. She lives in an ethnic isolate and is at the age of 32 tumor‐free and in comparatively good health.

Thus, taking all evidence together, both natural and medical radiation was clearly higher in CS than in Poland, although the difference in the annual doses received is perhaps only in the order of a few mSv. Could this affect the age of cancer (lymphoma) manifestation? Risk estimates at these low dose levels represent a challenge for epidemiology. The results of a number of studies of lymphoma risk after exposure to low dose ionizing radiation are controversial. In a recent European multicenter case–control study, the cumulative dose (median 2.25 mGy) was not positively associated with the lymphoma risk.^31^ However, the results of this cohort can hardly be extrapolated to the highly radiosensitive NBS homozygotes. Therefore, in our opinion, the most plausible explanation for the age difference in cancer manifestation between the CS and Polish patients is the different natural and medical radiation exposure. Today, this hardly plays a role, since in both countries NBS diagnosis is made shortly after birth and families are counseled accordingly.

Of greater relevance, however, is whether the increased cancer risk of heterozygotes in CS is a consequence of low additional radiation exposure. Worldwide, this would affect more than a million of heterozygotes.^8^ Since their cancer risk is increased, cancer screening, such as regular mammograms for early detection of breast cancer, would be indicated. However, could this itself not significantly increase the risk of cancer? This has previously been suggested for heterozygotes with mutations in the *ATM* gene, another autosomal recessive disease with a high sensitivity to radiation and a greatly increased risk of cancer. The discussion about this, especially with regard to alternatives to radiodiagnostics, has been going on for a long time^32^ and in our opinion is also relevant for *NBN* heterozygotes. To this end, a comparative study in both countries would be desirable in the context of personalized medicine.

## AUTHOR CONTRIBUTIONS

**Krystyna Chrzanowska:** Conceptualization (equal); data curation (equal); investigation (equal); project administration (equal); resources (equal); supervision (equal). **Eva Seemanova:** Conceptualization (equal); data curation (equal); investigation (equal); project administration (equal); resources (equal); supervision (equal). **Raymonda Varon:** Data curation (equal); investigation (equal); methodology (equal). **Martin Digweed:** Formal analysis (equal); funding acquisition (equal); resources (equal); writing – original draft (equal). **Dorota Piekutowska:** Investigation (equal); methodology (equal). **Karl Sperling:** Conceptualization (equal); formal analysis (equal); funding acquisition (equal); investigation (supporting); methodology (supporting); project administration (supporting); resources (equal); supervision (equal); writing – original draft (lead). **Pavel Seeman:** Data curation (equal); funding acquisition (equal); investigation (equal); project administration (equal); writing – original draft (equal).

## CONFLICT OF INTEREST

The authors have stated explicitly that there are no conflicts of interest in connection with this article.

## ETHICS STATEMENT

All procedures were performed in accordance with the ethical standards of the responsible committees: Ethical approval of the Ethics Committee of The Children's Memorial Health Institute, Warsaw and the Ethics Committee of the Second Medical School of the Charles University, Prague.

## Supporting information


**FIGURE S1** (A) Locations of NBN homozygotes from Poland to voivodships. No information was available for two sibs. (B) Locations of NBN homozygtes from the Czech Republic/Slovakia

**TABLE S1** Age difference at cancer manifestation between *NBN* homozygote sibs from CS and Poland
**TABLE S2** Age difference at cancer manifestation between unrelated NBN homozygotes from CS and Poland

## Data Availability

The data that supports the findings of this study are available in this article. Individual deidentified participant data will be shared by Krystyna H. Chrzanowska and Pavel Seeman.
