# Supplementary material for: The NBN founder mutation—Evidence for a country specific difference in age at cancer manifestation
Source: Cancer Rep (Hoboken). 2022 Aug 10;6(2):e1700. doi: 10.1002/cnr2.1700 (PMC9939984; doi:10.1002/cnr2.1700)
Supplement: Supplementary file 1 — FIGURE S1 (A) Locations of NBN homozygotes from Poland to voivodships. No information was available for two sibs. (B) Locations of NBN homozygtes from the Czech Republic/Slovakia [file CNR2-6-e1700-s002.pptx]

## Slide 1
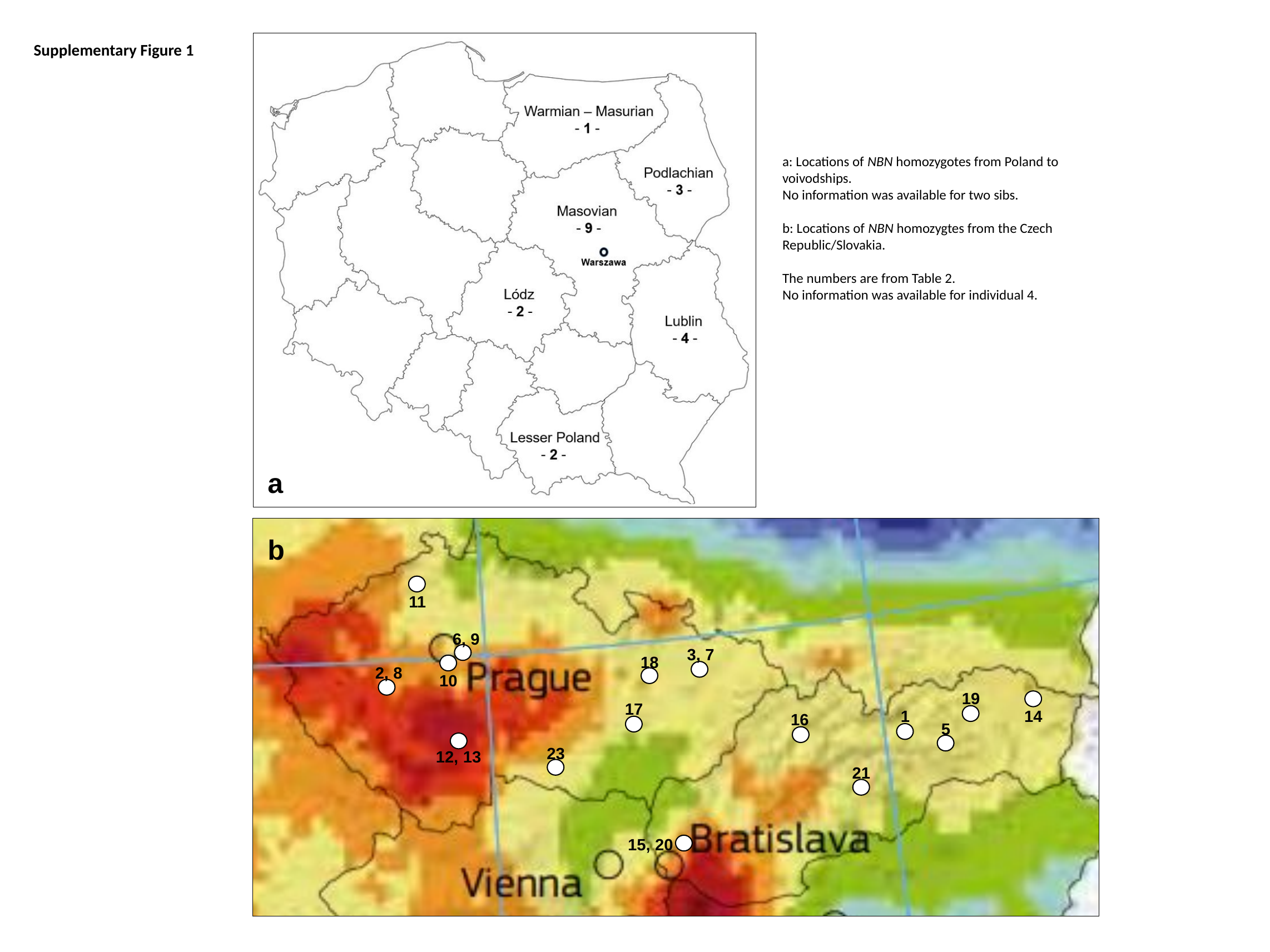

Supplementary Figure 1
a: Locations of NBN homozygotes from Poland to voivodships.
No information was available for two sibs.
b: Locations of NBN homozygtes from the Czech Republic/Slovakia.
The numbers are from Table 2.
No information was available for individual 4.
a
b
11
6, 9
3, 7
18
2, 8
10
19
17
1
14
16
5
23
12, 13
21
15, 20
