# Supplementary material for: The NBN founder mutation—Evidence for a country specific difference in age at cancer manifestation
Source: Cancer Rep (Hoboken). 2022 Aug 10;6(2):e1700. doi: 10.1002/cnr2.1700 (PMC9939984; doi:10.1002/cnr2.1700)
Supplement: Supplementary file 2 — TABLE S1 Age difference at cancer manifestation between NBN homozygote sibs from CS and Poland TABLE S2 Age difference at cancer manifestation between unrelated NBN homozygotes from CS and Poland [file CNR2-6-e1700-s001.docx]

Supplementary Table 1a

Age difference at cancer manifestation between *NBN* homozygote sibs from CS and Poland

| N | sex | Year of birth | cancer | Age at cancer manifest. | Age at death | Age difference at cancer manifestation |
| --- | --- | --- | --- | --- | --- | --- |
| 2 | m | 1970 | ALL | 10 | 10 | 9 |
| 8 | f | 1978 | ALL | 1 | 1 |  |
| 3 | f | 1971 | NHL | 18 | 22 | 16 |
| 7 | m | 1977 | NHL | 2 | 2 |  |
| 6 | m | 1975 | NHL | 24 | 29 | 13 |
| 9 | f | 1979 | Ewing Sarc. | 11 | 17 |  |

| 6 | f | 1979 | Lymphoma | 15 | 15 | 20 |
| --- | --- | --- | --- | --- | --- | --- |
| 8 | m | 1981 | ALL-T | 35 | 36 |  |
| Average difference at cancer manifestation (years) | | | | | | 14.5 |

Supplementary Table 1b

Age difference at cancer manifestation between unrelated *NBN* homozygotes from CS and Poland

| N | sex | | Year of birth | cancer | Age at cancer manifest. | Age at death | Age difference at cancer manifestation |
| --- | --- | --- | --- | --- | --- | --- | --- |
| 1 | f | | 1969 | NHL | 10 | 10 | 7 |
| 4 | f | | 1973 | Gonadobl. | 17 | 19 |  |
| 10 | f | | 1979 | Meningioma | 10 | (26)^1^ | 3 |
| 11 | m | | 1980 | ALL | 13 | (25)^1^ |  |
| 14 | f | | 1983 | Medullobl. | 7 | 7 | 9 |
| 16 | f | | 1985 | NHL | 16 | (20)^1^ |  |
| 17 | f | | 1986 | NHL | 7 | 10 | 2 |
| 18 | m | | 1988 | NHL | 9 | 10 |  |
|  | | | | | | | |
| 1 | m | 1962 | | T-NHL | 34 | 34 | 19 |
| 3 | f | 1978 | | B-NHL | 15 | 21 |  |
| 7 | m | 1980 | | DLBCL-1-5 | 11 | 29 | 5 |
| 10 | m | 1982 | | TLBL/ALL | 16 | 19 |  |
| 11 | m | 1983 | | T-NHL | 11 | 12 | 4 |
| 13 | m | 1985 | | B-NHL | 7 | 19 |  |
| 14 | m | 1985 | | Thyroid Ca | 20 | (35)^1^ | 12 |
| 15 | f | 1985 | | Medullobl. | 8 | 9 |  |
| 16 | m | 1986 | | HL | 12 | 14 | 3 |
| 17 | f | 1987 | | B-NHL | 9 | 9 |  |
| 18 | f | 1987 | | T-NHL | 19 | 21 | 10 |
| 19 | f | 1988 | | T-NHL | 24 | 24 |  |
| 20 | f | 1988 | | B-NHL | 12 | 13 | 3 |
| 22 | f | 1989 | | TLBL/ALL | 9 | 12 |  |
| Average difference at cancer manifestation (years) | | | | | | | 7.0 |

^1^age at last contact
